# Quantification of Antiviral Cytokines in Serum, Cerebrospinal Fluid and Urine of Patients with Tick-Borne Encephalitis in Croatia

**DOI:** 10.3390/vaccines10111825

**Published:** 2022-10-29

**Authors:** Snjezana Zidovec-Lepej, Tatjana Vilibic-Cavlek, Maja Ilic, Lana Gorenec, Ivana Grgic, Maja Bogdanic, Leona Radmanic, Thomas Ferenc, Dario Sabadi, Vladimir Savic, Zeljka Hruskar, Luka Svitek, Vladimir Stevanovic, Ljiljana Peric, Dubravka Lisnjic, Danijela Lakoseljac, Dobrica Roncevic, Ljubo Barbic

**Affiliations:** 1 Department of Immunological and Molecular Diagnostics, University Hospital for Infectious Diseases “Dr Fran Mihaljevic”, 10000 Zagreb, Croatia; 2 Department of Virology, Croatian Institute of Public Health,10000 Zagreb, Croatia; 3 Department of Microbiology, School of Medicine, University of Zagreb, 10000 Zagreb, Croatia; 4 Department of Epidemiology, Croatian Institute of Public Health, 10000 Zagreb, Croatia; 5 Clinical Department of Diagnostic and Interventional Radiology, Merkur University Hospital, 10000 Zagreb, Croatia; 6 Clinic for Infectious Diseases, Clinical Hospital Center Osijek, 31000 Osijek, Croatia; 7 Medical Faculty, Josip Juraj Strossmayer University of Osijek, 31000 Osijek, Croatia; 8 Laboratory for Virology and Serology, Poultry Center, Croatian Veterinary Institute, 10000 Zagreb, Croatia; 9 Department of Microbiology and Infectious Diseases with Clinic, Faculty of Veterinary Medicine, University of Zagreb, 10000 Zagreb, Croatia; 10 Primorje-Gorski Kotar County Teaching Institute of Public Health, 51000 Rijeka, Croatia

**Keywords:** tick-borne encephalitis, cytokines, cerebrospinal fluid, serum, urine

## Abstract

Background: Tick-borne encephalitis virus (TBEV) is one of the most significant arboviruses affecting the human central nervous system (CNS) in Europe. Data on cytokine response in TBEV infection are limited. Methods: We analyzed the cytokine response in serum, cerebrospinal fluid (CSF) and urine samples of patients with TBE. The control group consisted of patients with ‘febrile headache’ who had normal CSF cytology. The panel included 12 cytokines: TNF-α, IL-6, Th1 (IL-2, IFN-γ), Th2 (IL-4, IL-5, IL-13), Th9 (IL-9), Th17 (IL-17A, IL-17F), Th22 (IL-22) cytokines and IL-10. Results: TBE patients were more likely to have increased levels of IL-6 and IFN-γ in CSF compared to controls (85.7% vs. 58.8% and 85.7% vs. 47.1%, respectively). However, concentrations of IL-6 (the most abundant cytokine in the CSF of both groups), IL-10 and IL-9 were lower in TBEV patients compared with controls, but the difference was statistically significant for IL-9 only (*p* = 0.001). By analyzing the cytokine levels in different clinical samples, all measured cytokines were detected in the serum, with the highest concentrations found for IFN-γ, TNF-α, IL-10, IL-17F and IL-22. Higher concentrations of cytokines in the CSF compared with serum were observed for IL-5, IL-6 and IL-22. All cytokines except IL-13 were detectable in urine but in a small proportion of patients, except for IL-22, which was detectable in 95.8% of patients. Conclusions: Cytokine composition in different clinical samples of TBE patients reveals a different network of early innate immune response cytokines, Th1, Th2, Th9, Th22, Th17 and anti-inflammatory cytokines.

## 1. Introduction

Tick-borne encephalitis virus (TBEV) is a positive sense, single-stranded RNA virus that belongs to the family *Flaviviridae*, genus *Flavivirus*, tick-borne encephalitis serocomplex. There are three subtypes of TBEV: European, Far Eastern and Siberian, each with a unique geographical distribution, tick vector and clinical presentation [1]. The virus is transmitted mainly through a bite of TBEV-infected ticks (*Ixodes ricinus*, *Ixodes persulcatus*); however, the infection may also occur as a result of consuming raw milk or unpasteurized dairy products from infected animals (goat, sheep and cow) [2].

TBEV is one of the most important arboviruses affecting the human central nervous system (CNS) in Europe. The disease is endemic in 27 central, north and eastern European countries, and a rise in morbidity was noted [1]. Between 2000 and 2019, the mean incidence rate of TBE was 3.27, whereas the age-adjusted mean incidence rate was 2.19 per 100,000 population size [3]. New endemic areas have been discovered in recent years as a result of TBE emergence in previously unaffected areas [4]. The majority of cases in Europe occur in June and July when the tick activity peaks; however, the transmission season may last from April to November [3]. In Croatia, TBE is endemic in northwestern and northeastern continental regions, between the Rivers Sava and Drava. Endemicity is highest in northwestern counties, with a mean incidence of 3.61–6.78/100,000 inhabitants. From 2009 to 2021, between four and forty-five cases of TBE were reported annually, with an incidence of 0.14–1.05 per 100,000 inhabitants [5,6]. In recent years, TBEV emerged in some local areas in the mountainous region of Gorski Kotar [4]. The virus has also been found in small foci in the Middle and South Croatian littoral [5]. 

The exact mechanism of TBEV neuroinvasion is not clear. The virus could enter the CNS without disrupting the blood–brain barrier since its permeability results from cytokine release in response to viral replication inside the brain [1]. The clinical spectrum of TBE varies from mild meningitis to severe meningoencephalitis with or without paralysis [7]. Typically, infection with the European TBEV has a biphasic course. The first phase corresponds to viremia and is characterized by fever, headache, malaise and myalgia. After a short improvement, the second phase occurs with the CNS symptoms. In contrast, Far Eastern and Siberian TBE usually have a monophasic course. The case-fatality rate is 0.5–2% for the European subtype and up to 35% for the Far Eastern subtype [8].

Diagnosis of TBE can be confirmed by the detection of TBEV RNA and/or antibodies. IgM and IgG-specific TBEV antibodies are present in the serum and cerebrospinal fluid (CSF) of TBE patients. TBEV-specific IgM, and frequently IgG antibodies, are detectable in serum at the onset of neurological symptoms. In the CSF, IgM begins to rise within six days, peaking at around 14 days following the onset of CNS symptoms [9]. IgM antibodies may persist for several months or even years in some rare cases; IgG avidity (affinity of IgG antibodies to bind the antigen) can differentiate recent (low IgG avidity) from past infection (high IgG avidity) in these cases [10]. TBEV RNA can be detected in the blood during the first phase of the disease and rarely in the CSF [11]. 

The contribution of cytokines, chemokines and other biological response modifiers to the pathogenesis of TBE has been evaluated in a number of studies that investigated the intrathecal synthesis of these molecules in the meningoencephalitic phase of the disease and evaluated their possible role as biomarkers of disease severity and/or outcome [12,13,14,15,16,17,18,19,20,21,22,23,24]. 

Early studies focused on the role of chemokines in TBE, particularly CXCL10, a non-ERL chemokine lacking the Glu-Leu-Arg tripeptide motif that is responsible for the recruitment of activated Th1 lymphocytes to sites of inflammation. CXCL10 concentration gradient between CSF and serum of TBE patients suggested the importance of this chemokine in the recruitment of CXCR3-expressing T-cells, particularly memory CD45RO+CD4+ T-cells, into the CNS [10]. Enrichment of the CSF with CD4+ T-cells in TBE was confirmed more recently by Toczylowski et al. (2020) [13]. 

Subsequent studies showed significant differences in the concentrations of various chemokines, including CCL2, CXCL5, CXCL10, CXCL11, CXCL12, CXCL13, macrophage migration inhibitory factor (MIF), IL-8, CCL7 in the CSF and CXCL10, CXCL13 and MIF in the serum of TBE patients, usually in comparison with patients diagnosed with other inflammatory CNS diseases [14,15,16,17,18].

Studies on cytokine biology in TBE showed increased serum levels of IL-1α, TNF-α, IL-6, IL-8, IL-12, IL-15, IL-18 and IFN-γ, as well as increased concentrations of IL-1β, IL-16, Th17 cytokines (IL-17A, IL-17F) and Th22 cytokine IL-22 in the CSF of TBE patients in comparison with controls [15,16,19,20]. The concentration of IL-17F correlated with the neutrophil count, and IL-16 CSF concentrations correlated positively with the CSF lymphocyte count in TBE patients [16,17]. 

Most recently, Bogovič et al. (2022) characterized cytokine and chemokine responses in the serum of 88 TBE patients during the first disease stage and in the serum and CSF during the second disease stage [21]. Distinct patterns of cytokine/chemokine expression in the first and second stages of TBEV infection have been described with initial expression of CXCL11, CXCL13, BAFF, IL-27 and IL-4 followed by an expression of pro-angiogenic growth factors, VEGF-A and GRO-α, in the serum at the second stage of the disease. During the meningoencephalitic stage of TBE, expression of IL-6, IFN-γ, CXCL10, CCL19 and APRIL in the CSF has been shown.

Data from the abovementioned studies show the complexity of immunopathogenic mechanisms in TBEV infection that appear to be mediated by a variety of biological response modifiers in different clinical samples and stages of infection. Interestingly, the expression of cytokines and chemokines in other clinical samples, such as urine, has not been investigated so far in TBE. 

Cytokines and chemokines were also evaluated as markers of disease severity, e.g., as predictors of moderate to severe encephalitis vs. predominantly meningeal symptoms and predictors of TBE meningoencephalitis vs. meningitis in both adults and pediatric patients [13,22,23,24,25]. These data suggest a possible use of several cytokines and chemokines as biomarkers of disease severity in TBE, but due to the heterogeneity of the available data, a minimal set of optimal biomarker candidates has yet to be determined. 

The aim of this study was to compare the expression of 12 cytokines, including those associated with innate and early pro-inflammatory immune responses (TNF-α, IL-6), Th1 (IL-2, IFN-γ), Th2 (IL-4, IL-5, IL-13), Th9 (IL-9), Th17 (IL-17A, IL-17F), Th22 (IL-22) cytokines and the anti-inflammatory cytokine IL-10 in different clinical samples (including CSF, serum and urine) of adults with TBE. Since there are no data on the cytokine expression in urine samples of patients with neuroinvasive arbovirus infections, urine samples were also selected for cytokine determination.

## 2. Materials and Methods

### 2.1. Patients

A total of 48 patients with confirmed TBEV infections detected from 2017 to 2022 were included in the study. A control group consisted of 17 patients with ‘febrile headache’ who had normal CSF cytology (<5 cells/μL). CSF (*n* = 28), serum (*n* = 46) and urine (*n* = 24) from patients with TBE and CSF (*n* = 17) from control group were available for the cytokine determination. The study included all patients with confirmed TBE during the abovementioned transmission seasons and the controls that met the selection criteria sampled within the identical time frame. Median sampling time in patients with TBE and the control group was 11 (IQR 8–15) days and 4 (IQR 2–6), respectively, after disease onset. 

TBE was confirmed in all patients according to the criteria of the European Center for Disease Prevention and Control (ECDC) [26] by detection of TBEV IgM and IgG in serum and IgM antibodies in the CSF. Samples with detected cross-reactive flavivirus antibodies were further confirmed by a virus neutralization test (VNT). Since cases of TBE without pleocytosis were described [27], CSF samples of all participants from the control group were also tested for TBEV RNA and IgM antibodies and showed negative results.

### 2.2. Methods

#### 2.2.1. Serology

TBEV IgM and IgG antibodies were detected using a commercially ELISA (Euroimmun, Lübeck, Germany), and interpreted as follows: IgM (ratio) < 0.8 negative, 0.8–1.1 borderline, >1.1 positive; IgG (RU/mL) < 16 negative, 16-22 borderline, >22 positive. For the VNT, the TBEV Ljubljana strain (provided by the European Virus Archive goes Global; EVAg project) was used as an antigen for VNT. The virus titer (median tissue culture infectious dose; TCID_50_) was determined on day 5 after inoculation using the Reed and Muench formula. Serum samples were heat-inactivated (30 min/56 °C), and serial two-fold dilutions starting at 1:5 were prepared in duplicate in 96-well microtiter plates using a Dulbecco’s Modified Eagle Medium (DMEM; Lonza, Basel, Switzerland). An equal amount (25 μL) of inactivated serum dilutions and 100 TCID_50_ of TBEV were mixed and incubated for 1 h at 37 °C. In the final step, 50 μL of 2 × 10^5^ Vero E6 cells/mL in DMEM with 5% of heat-inactivated fetal calf sera (Capricorn Scientific, Ebsdorfergrund, Germany) were added to each well. In order to ensure optimal testing results, virus suspensions used in each run (incubated at 37 °C with CO_2_ for 1 h) were back titrated in four tenfold dilutions: 100, 10, 1 and 0.1 TCID_50_. Positive and negative control were included in each plate. The plates were incubated at 37 °C with CO_2_ for five days. The plates were examined for the cytopathic effect (CPE) starting from the third day of incubation. The optimal time for reading the results was when CPE was visible in all six wells of 100 and 10 TCID_50_ and two to three wells containing 1 TCID_50_ of virus in back titration. Antibody titer was defined as the reciprocal value of the highest dilution of the serum that showed 100% neutralization. Titer ≥ 1:10 was considered positive [4].

#### 2.2.2. Cytokine Quantification

Concentrations of selected cytokines in the serum, CSF and urine were determined by multiplex bead-based flow cytometry assay (LEGENDplex Human Th cytokine panel, BioLegend, San Diego, CA, USA) that allows simultaneous quantification of 12 cytokines on FACS Canto II instrument (Beckton Dickinson, Franklin Lakes, NJ, USA) [28]. The samples were stored at −80 °C in aliquots in order to avoid freeze-and-thaw cycles. The cytokine panel included mediators of innate and early immune responses (IL-6 and TNF-α), Th1 cytokines (IFN-γ and IL-2), Th2 cytokines (IL-4, IL-5, IL-9 and IL-13), Th17 cytokines (IL-17A, IL-17F and IL-22) and the anti-inflammatory cytokine IL-10. The sensitivity of the assay, calculated as minimum detectable concentration ± 2 standard deviations for individual cytokines was: IL-6 (1.0 + 0.8 pg/mL), TNF-α (0.7 + 0.5), IFN-γ (1.1 + 0.7), IL-2 (1.4 + 0.4), IL-4 (1.0 + 0.8), IL-5 (1.2 + 1.3 pg/mL), IL-9 (1.7 + 1.4), IL-13 (1.4 + 0.7), IL-17A (1.9 + 0.6), IL-17F (0.8 + 0.7), IL-22 (1.5 + 0.5) and IL-10 (0.7 + 0.4). Cytokine concentrations in the serum of healthy controls (*n* = 16) were: IL-6 (mean 12.9 pg/mL, range not detected (ND)-18.4 pg/mL, detectable in 56% of samples); TNF-α (5.9, ND-14.0, detectable in 50% of samples); IFN-γ (mean 11.5, range ND-39.4, detectable in 63% of samples); IL-2 (mean 39.1, range ND-79.4, detectable in 25% of samples); IL-4 (mean 18.1, ND-56.5, 44% of samples); IL-5 (mean 3.9, range non-detectable, ND-12.7, detectable in 69% of samples); IL-9 (mean 3.9, range ND-18.4, detectable in 25% of samples); IL-13 (mean 5.1, range 1.4-17.3, detectable in all samples); IL-17A (not detectable) and IL-17F (mean 29.0, range ND-107.0, detectable in 50% of samples); and IL-22 (mean 6.4, range ND-15.2, detectable in 38% of samples) and IL-10 (mean 1.1, range ND-1.3, detectable in 19% of samples) [22,29]. 

#### 2.2.3. Statistical Analysis

Descriptive methods were used to summarize information on the study participant’s age, gender and clinical presentation. Categorical variables are presented as frequencies and percentages, while numerical variables are presented as medians and interquartile ranges (IQR). Non-parametric tests were used for the data with non-normal distribution or when the outcome had clear limits of detection. Mann–Whitney U test was used to compare the age of patients with confirmed TBEV infection and controls, and Kruskal–Wallis test to compare the age of patients by clinical presentation. The proportion of serum, CSF and urine samples with positive cytokine response was calculated, as well as the median and IQR of the cytokine concentration for positive samples. The concentrations of the measured cytokines in paired serum, CSF and urine samples were compared using the Friedman test. Crude odds ratios (cOR) and adjusted odds ratios (aOR) for age and sex are calculated to measure the association between the positive response of investigated cytokines and TBEV diagnosis. The level of significance is set to *p* values < 0.05. Statistical analysis was performed using Stata, version 17 software.

## 3. Results

Demographic and clinical characteristics of patients with TBE and the control group are presented in Table 1. The median age of TBE patients was 49 years, ranging from 15 to 85, with IQR = 36–65 years. In the tested group (TBE patients; *n* = 48), there were 34 (70.8%) males (median age 47.8, IQR = 66–61 years) and 13 (27.1%) females (median age 50.8, IQR = 35.5–67.5 years). Gender and age were unknown for one patient. Patients presented with following diagnoses: meningitis (*n* = 24), meningoencephalitis (*n* = 15) and ‘febrile headache’ (*n* = 9). There was no difference in age among patients with different clinical diagnoses (*p* = 0.86). In the control group (*n* = 17), there were eight (47.1%) males (median age 65.2, IQR = 57.5–75.5 years) and nine (52.9%) females (median age 71, IQR = 63.5–70 years). Participants in the control group presented with ‘febrile headache’ (*n* = 6) and ‘febrile headache’ with impaired consciousness (*n* = 11). The control study participants were older than TBE patients (median age 61, IQR = 50–71, *p* = 0.04). 

In the patients with confirmed TBE, the median CSF leukocyte count was 186 (IQR = 105–323), with mononuclear predominance. In the control group, the median leukocyte count was 1.7 (IQR = 1–2.5) (Table 2). The median concentration of proteins in both patient groups (0.756 g/L in TBE patients vs. 0.464 g/L in controls) was increased. 

The proportion of TBE patients and control samples with positive cytokine response in the CSF is presented in Table 3. TBEV cases were more likely to have increased levels of IL-6 and IFN-γ in CSF comparing to controls (crude OR = 4.2, *p* = 0.04 and OR = 6.7, *p* = 0.006, respectively). However, when adjusted by age and gender, the difference did not reach statistical significance (adjusted OR = 3, *p* = 0.19; adjusted OR = 4.5, *p* = 0.05). Expression of IL-9 and IL-22 exhibited an opposite pattern with higher proportions of individuals with detectable cytokines in the control group (82.2% and 94.1%, respectively) compared with TBEV cases (67.9% and 71.4%, respectively), but the difference did not reach statistical significance.

Concentrations of cytokines in the CSF of TBEV patients and controls are presented in Table 4. Concentrations of IL-6 (the most abundant cytokine in the CSF of both groups), IL-10 and IL-9 were lower in TBEV patients compared with controls, but the difference was statistically significant for IL-9 only (*p* = 0.001). Concentrations of IL-5, IFN-γ and IL-22 were higher in the TBEV group compared with controls. Th2 cytokine IL-13, as well as IL-17A, were undetectable in the CSF. Several cytokines, including IL-2, TNF-α, IL-17F and IL-4, were detectable in the CSF of a small proportion of TBEV patients only (1–3 patients for each cytokine). 

**Figure 1:**
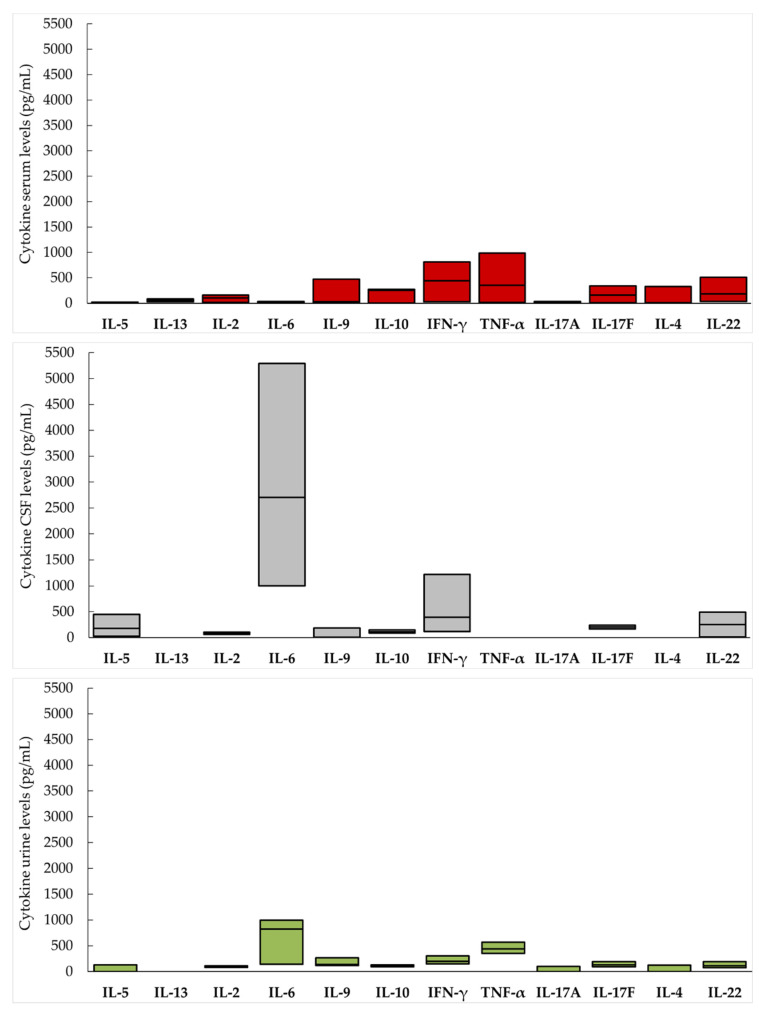
Cytokine levels in serum, cerebrospinal fluid (CSF) and urine of patients with TBE.

All measured cytokines were detected in the serum, with the highest proportion of patients with detectable cytokines reported for IL-6, IL-9, IL-22, IFN-γ, TNF-α, IL-17A and IL-17F (between 39.1 and 60.9%, Figure 1). The highest serum concentrations were shown for IFN-γ (median 440.3 pg/mL), TNF-α (median 357.2 pg/mL), IL-10 (257.0 pg/mL), IL-17F (160.4 pg/mL) and IL-22 (185.6 pg/mL). 

Higher concentrations of cytokines in the CSF compared with serum, indirectly suggesting an intrathecal synthesis, were observed for several cytokines, including IL-5 (CSF median 181.1 pg/mL vs. 12.4 pg/mL), IL-6 (2705.7 vs. 23.9 pg/mL) and IL-22 (254.5 vs. 185.6 pg/mL). 

All cytokines except IL-13 were detectable in urine but in a small proportion of patients, except for IL-22, which was detectable in 95.8% of patients. The highest cytokine concentrations in urine were observed for IL-6 (median 822.4 pg/mL) and TNF-α (median 438.0 pg/mL) and were higher compared with serum samples (Figure 1). 

## 4. Discussion

The results of this study represent the first systematic comparison of CSF, serum and urine concentrations of cytokines in patients with TBE. All measured cytokines were detectable in the serum and in urine (except IL-13) in various proportions of patients. IL-22 was present in the urine of almost all TBE patients, with the highest concentrations of IL-6 and TNF-α in this clinical sample. High intrathecal expression of IL-5, IL-6 and IL-22 in TBE patients shows that local immune responses to TBEV involve a network of diverse cytokines associated with early innate and proinflammatory cytokines, Th2 and Th22 cytokines. Significantly lower concentrations of IL-9 in the CSF of TBE patients in comparison with patients presenting with ‘febrile headache’ or impaired consciousness as controls.

IL-6 exhibits a dual role in the biology of CNS. By using a classical signaling pathway involving membrane IL-6 receptor, it exhibits a neuroprotective role by promoting differentiation of oligodendrocytes, regeneration of peripheral nerves and acting as a neurotrophic factor. The trans-signaling pathway involves the formation of a complex between sIL-6R and IL-6 that binds to the gp130 subunit expressed on various types of cells that would be unresponsive to this cytokine, such as neurons and astroglia. Trans-signaling is typically associated with neuronal degeneration and differentiation of microglia into a proinflammatory phenotype. The role of IL-6 as a biomarker in neuroinflammatory CNS diseases has been extensively evaluated, but the data on CNS viral infections are more limited [30]. In this study, we showed the expression of IL-6 in the majority of TBE patients and demonstrated a high intrathecal concentration of IL-6. These results are in concordance with a recent study by Bogovič et al. (2022), which also demonstrated the high-level intrathecal synthesis of IL-6 in the meningoencephalitic stage of the disease [21]. In addition, our study provided the first experimental evidence of high-level expression of IL-6 in the urine of TBE patients. We reported significantly higher concentrations of IL-6 in the CSF of patients with West Nile virus (WNV) neuroinvasive disease (WNND), including those with severe clinical presentation, compared with serum [31,32]. These results suggest a possible common pattern of local cytokine response to flaviviruses in the CNS. In addition, high intrathecal concentrations of IL-6 were shown in three patients with Toscana virus (TOSV) neuroinvasive disease, suggesting that more extensive analysis of the role of IL-6 in CNS infections with other RNA viruses might help elucidate possible common immunopathogenic mechanisms in these events [33].

High intrathecal concentrations of IL-5, a Th2 cytokine that is associated with a pattern of cytokine responses that is unfavorable in viral infections, as well as the lack of obvious CSF/serum ratio in the concentration in IFN-γ and IL-2 as signature Th1 cytokines, suggests that polarization towards Th2 responses contributes to the pathogenesis of TBE. Although the Th2 cells that are characterized by the synthesis of IL-4, IL-5 and IL-13 are usually considered relevant for humoral immunity, protection from helminth infection and pathogenesis of the allergic inflammatory disease, the recent discovery of memory-type pathogenic Tpath2 cells by Nakayama et al. (2017) re-defined our understanding of IL-5 [34]. Unique features of Tpath2 cells include high-level expression of the receptor for IL-33 that is synthesized following tissue damage and production of IL-5 in high quantities. The possible contribution of these cells to the immunopathogenesis of CNS diseases and the role of IL-5 in this context need to be characterized in the future. 

IL-9 is a pleiotropic cytokine that exhibits a complex pattern of proinflammatory and anti-inflammatory effects on various cell types, depending on the local cytokine and signaling molecule microenvironment. It can be synthesized by Th9 cells, CD8+ T-cells, B-cells, NKT cells, innate lymphoid cells, mast cells, γδ T cells and granulocytes. The main biological activities of IL-9 include expansion of Th17 cells, enhancement of the immunosuppressive function of regulatory T-cells, activation of dendritic cells towards Th2 type responses, activation of mast cells and NKT cells as well as modulation cytotoxic T-cell activities [35]. Ding et al. (2015) demonstrated high-level expression of IL-9 receptor (IL-9R) on astrocytes, oligodendrocyte progenitor cells (OPG), oligodendrocytes and microglia cells, showing its important role in the biology of the CNS [36]. In addition, IL-9 increases the expression of CXCL9, CCL29 and MMP3 in primary astrocytes and promotes the proliferation and differentiation of OPC in combination with IFN-γ in vitro. In addition to its well-established role in the pathogenesis of asthma and chronic inflammatory diseases, the role of IL-9 in the pathogenesis of inflammatory CNS diseases, including multiple sclerosis and experimental autoimmune encephalomyelitis (EAE), has been extensively studied. IL-9 was shown to have a pathogenic role in EAE mediated by promoting T-cell activation and differentiation, but more recent studies suggest an important role of IL-9 in controlling the pathogenic inflammation mediated by Th17 cells [37]. Therefore, the precise role of this cytokine in the pathogenesis of CNS diseases remains unknown. Higher concentrations of IL-9 in the CSF of patients with febrile headache or impaired consciousness compared with TBE patients suggests that, despite normal CSF cytology, patients from the control group experienced local inflammatory reactions. Increased protein CSF concentration in the control group supports this hypothesis.

IL-22 is a cytokine that belongs to the IL-10 cytokine family and plays an important role in mucosal immunity by promoting the repair and regeneration of epithelial cells and by downregulating inflammatory immune responses [38]. It is mainly synthesized by Th22 cells but can also be produced by ILC3, γδ T, iNKT, Th17 and granulocytes. Expression of the IL-22 receptor is restricted mainly to epithelial cells and fibroblasts. The role of IL-22 in the pathogenesis of autoimmune and infectious diseases, as well as in malignancies, has been extensively studied. Recently, Eken et al. (2021) showed that IL-22 overexpression significantly decreased EAE scores and demyelination and reduced infiltration of IFN-γ+IL-17A+Th17 cells into the CNS of mice [39]. However, due to the heterogeneity of the literature data and the lack of the possible role of IL-22 in CNS viral infections is currently unknown.

The role of IL-22 in the pathogenesis of flavivirus neuroinvasive infections was investigated a knock-out mice Il22(-/-), showing that IL-22 signaling exacerbated lethal WNV encephalitis by promoting neuroinvasion in a process mediated by a chemokine receptor CXCR2 responsible for the migration of neutrophils into the brain [40]. IL-22 was also detected in the CSF of patients with WNV and TOSV neuroinvasive diseases, suggesting a possible role of these cytokines in the neuropathogenesis of various CNS infections [28,29,30]. Grygorczuk et al. (2018) demonstrated significantly increased concentrations of IL-22 in the CSF of TBE patients [14]. Our results showed the presence of IL-22 in all clinical samples from TBE patients (serum, CSF, urine) as well as a serum/CSF gradient of IL-22, suggesting intrathecal synthesis of this cytokine that might contribute to the pathogenesis of TBE. 

IL17A and IL-17F are members of the IL-17 cytokine family that share the highest amino acid sequence homology (55%) and act as homodimers or IL-17A/IL-17F heterodimers. Biological features of the two cytokines appear to be distinct, with IL-17F being best characterized as a mediator of mucosal immunity and IL-17A as an important mediator of inflammatory reactions and autoimmunity [41]. 

In our study, IL-17A was not detected in the CSF and urine (except in one patient) of TBE patients with very low serum levels of these cytokines. Expression of IL-17F was detected in the CSF of only two TBE patients but was detectable in the serum and urine of TBE patients. IL-17F was undetectable in the CSF of controls. The expression pattern of IL-17F and IL-17A in the serum TBE patients is consistent with the literature data in healthy controls [29]. Grygorczuk et al. (2018) analyzed IL-8, IL-17A, IL-17F and IL-22 patterns in a small cohort of TBE patients (*n* = 36 for IL-8, *n* = 15 for other), non-TBE aseptic meningitis patients (*n* = 6) and non-meningitis controls (*n* = 7) [16]. In contrast to our results, significantly increased concentrations of IL-17A and IL-17F in CSF of TBE patients, as well as upregulation of IL-17A in the serum, were shown [16]. In addition, CSF concentrations of IL-17 and CXCL1 correlated with neutrophil counts but not with disease severity. Pietikäinen et al. (2016) showed increased concentrations of IL-17 in the CSF of TBE patients in comparison with neuroborreliosis, but the results are difficult to interpret due to the lack of separate quantification of IL-17A and IL-17F [42]. The differences between our results and the previously reported literature data on Th17 cytokines in TBE could possibly be attributed to the different timelines of CSF collection during the presentation to care but need to be investigated further. 

The majority of the literature data on the role of Th17-type cytokines in other neuroinvasive flavivirus infections comes from studies in animal models of WNV infection and from studies in human WNND. Acharya et al. (2016) showed that IL-17A-knock out mice (Il17a-/-) are more susceptible to WNV infection and exhibit increased kinetics of viral replication compared with wild-type animals [43]. In addition, treatment of WNV-infected mice with recombinant IL-17A up to day 6 after infection reduces the viral replication and increases survival suggesting a protective effect of this cytokine in WNV infection. The results of our previous studies showed very low serum levels of IL-17A and the lack of IL-17A in the CSF of human WNND patients suggesting that the downregulation of Th17-mediated immunity might contribute to the pathogenesis of this disease [31,32]. The expression of IL-17A in TBE patients observed in this study is in concordance with the reported data in WNND, suggesting a common cytokine pattern of IL-17A expression in neuroinvasive flavivirus infections that needs to be investigated further. Lack of IL-17A and IL-17F expression in the CSF was also described in a report of three cases with TOSV neuroinvasive disease, but these data need to be substantiated in a much larger dataset [33]. 

The literature data on the expression of cytokines in the urine of patients with CNS infections and comparisons with CSF and serum concentrations are scarce. Salgado et al. (2019) failed to detect significant amounts of cytokines in the urine of children with Zika virus-associated encephalitis [44]. This study presents the first experimental evidence on the expression of cytokines in the urine of TBE patients. The consistent presence of IL-22 in the urine of almost all patients, as well as the fact that concentrations of IL-6 and TNF-α in urine have been higher compared with CSF and serum, clearly suggest a possible use of these non-invasive samples in immunological studies of viral CNS infection. 

Several limitations of our research need to be taken into account when interpreting this data. Analysis of cytokines in the CSF of TBE patients is limited to single time-point measurements. In addition, access to CSF from healthy persons is exceptionally restricted. Both limitations are related to obvious ethical reasons. Therefore, cytokine concentrations in the CSF can be only compared with patients that present to care with initial symptoms consistent with possible neuroinvasive infections that are subsequently excluded. Expression of cytokines in humans is regulated by individual host determinants such as age, sex, genetic background (single nucleotide polymorphisms in the cytokine genes) and chronic diseases (infectious and non-infectious) that also need to be taken into account when interpreting the data.

## 5. Conclusions

In conclusion, cytokine composition in different clinical samples of TBE patients (CSF, urine, serum) reveals a complex but different network of early innate immune response cytokines, Th1, Th2, Th9, Th22, Th17 and anti-inflammatory cytokines. These results provide a new insight into the pathogenesis of TBE that need to be investigated further. The precise nature of IL-5 and IL-9 to the pathogenesis of TBE needs to be clarified, possibly in animal models.

## Figures and Tables

**Table 1:** Demographic structure and clinical characteristics of patients with TBE and control group.

Group	Characteristic	N Tested (%)	Median, YRS (IQR)
Patients with TBE	Gender		
	Male	34 (72.2)	47.8 (36–61)
	Female	13 (27.8)	50.8 (35.5–67.5)
	Clinical presentation		
	Meningitis	25 (50.0)	48.5 (36–63.5)
	Meningoencephalitis	15 (31.3)	49.0 (36.0–58.0)
	‘Febrile headache’	9 (18.7)	51.0 (26.0–62.0)
Control group	Gender		
	male	8 (47.1)	65.2 (57.5–75.5)
	Female	9 (52.9)	71 (63.5–70)
	Clinical presentation		
	‘Febrile headache’	6 (35.3)	43.6 (32–56)
	‘Febrile headache’, impaired consciousness	11 (64.7)	67 (61–76)

IQR = interquartile range.

**Table 2:** Laboratory findings in CSF of patients with TBE and control group.

Group	Parameter	Median	IQR	Reference Range
Patients with TBE	Leukocyte count (cells/μL)	186	105–323	0–5
	Proteins (g/L)	0.756	0.624–0.800	0.170–0.370
	Glucose (mmol/L)	3.05	2.93–3.30	2.45–3.33
	Neutrophils (%)	36	18–72	
	Lymphocytes (%)	56	31–79	100
Control group	Leukocyte count	1.7	1–2.5	0–5
	Proteins (g/L)	0.464	0.333–0.591	0.170–0.370
	Glucose (mmol/L)	3.70	3.36–4.35	2.45–3.33

Group

Parameter

Median

IQR

Reference Range

**Table 3:** Proportion of cytokine detection in CSF of TBEV patients and controls.

	TBEV Patients (N = 28)		Controls (N = 17)					
Cytokine (CSF)	N (%) Positive	95% CI	N (%) Positive	95% CI	cOR (95% CI)	*p*	aOR (95% CI)	*p*
IL-5	4 (14.3)	4.0–32.7	1 (5.9)	0.1–28.7	2.7 (0.2–139.4)	0.38	1.2 (0.1–15.3)	0.89
IL-13	0 (0)	0–12.3 *	0 (0)	0–19.5 *	NA	NA	NA	NA
IL-2	3 (10.7)	2.2–28.2	0 (0)	0–19.5 *	NA	NA	NA	NA
IL-6	24 (85.7)	67.3–95.8	10 (58.8)	32.9–81.5	4.2 (0.8–23.5)	0.04	3.0 (0.6–16.0)	0.19
IL-9	19 (67.9)	47.6–84.1	15 (88.2)	63.5–98.5	0.3 (0.03–1.7)	0.1	0.2 (0.02–1.2)	0.08
IL-10	10 (35.7)	18.6–55.9	6 (35.3)	14.2–61.7	1.02 (0.2–4.4)	0.97	0.6 (0.1–2.6)	0.48
IFN-Ƴ	24 (85.7)	67.3–95.8	8 (47.1)	22.9–72.2	6.7 (1.3–37.1)	0.006	4.5 (0.97–20.9)	0.05
TNF-α	1 (3.6)	0.1–18.3	0 (0)	0–19.5 *	NA	NA	NA	NA
IL-17A	0 (0)	0–12.3 *	0 (0)	0–19.5 *	NA	NA	NA	NA
IL-17F	2 (7.1)	0.1–23.5	0 (0)	0–19.5 *	NA	NA	NA	NA
IL-4	1 (3.6)	0.1–18.3	0 (0)	0–19.5 *	NA	NA	NA	NA
IL-22	20 (71.4)	51.3–86.8	16 (94.1)	71.1–98.8	0.2 (0.00–1.4)	0.06	0.2 (0.02–2.0)	0.16

* One-sided 97.5% confidence interval; CI = confidence interval; cOR = crude odds ratio; aOR = adjusted odds ratio; NA = not applicable.

**Table 4:** Cytokine levels in CSF of TBEV patients and controls.

Cytokine	TBEV Patients		Controls		
	Median (pg/mL)	IQR	Median (pg/mL)	IQR	*p* *
IL-5	181.1	25.4–450.3	122.3	NA	NA
IL-13	NA	NA	NA	NA	NA
IL-2	81.5	61.7–106.0	NA	NA	NA
IL-6	2705.7	1000.4–5293.6	3224.8	1247.8–6032.2	0.64
IL-9	7.4	3.9–186.9	242.9	171.4–352.9	0.0001
IL-10	113.1	86.6–150.9	121.0	91.4–128.5	0.81
IFN-γ	394.3	120.4–1221.5	206.5	180.5–337.1	0.36
TNF-α	1246.2	1246.2–1246.2	NA	NA	NA
IL-17A	NA	NA	NA	NA	NA
IL-17F	203.8	166.8–240.8	NA	NA	NA
IL-4	241.0	241.0–241.0	NA	NA	NA
IL-22	254.5	11.8–491.7	172.7	110.3–201.7	0.31

* Mann–Whitney U test; IQR = interquartile range; NA = not applicable.

## Data Availability

Not applicable.
